# P-2051. Assessing SARS-CoV-2 Correlates Protection from Exposed Household Members: A Prospective Cohort Study (AB-PROTECT Study)

**DOI:** 10.1093/ofid/ofae631.2207

**Published:** 2025-01-29

**Authors:** Christopher Kandel, Maureen Taylor, Karen Colwill, Queenie Hu, Andrea Llanes, Gloria Crowl, Helen Deborah Elliott, Wang Kei Shi, Angel Li, Maxime Lefebvre, Yaejin Lee, Alexandra Kurtesi, Freda Qi, Melanie Delgado-Brand, Tulunay Tursun, Geneviève Mailhot, Robert Kozak, Janine McCready, Jeff Powis, Anne-Claude Gingras, Allison McGeer

**Affiliations:** Toronto East General Hospital, Toronto, Ontario, Canada; Michael Garron Hospital, Toronto, Ontario, Canada; Lunenfeld-Tanenbaum Research Institute, Toronto, Ontario, Canada; Lunenfeld-Tanenbaum Research Institute, Toronto, Ontario, Canada; Michael Garron Hospital, Toronto, Ontario, Canada; Michael Garron Hospital, Toronto, Ontario, Canada; Michael Garron Hospital, Toronto, Ontario, Canada; Michael Garron Hospital, Toronto, Ontario, Canada; Sinai Health System, Toronto, Ontario, Canada; Sinai Health, Toronto, Ontario, Canada; University of Toronto, Toronto, Ontario, Canada; Lunenfeld-Tanenbaum Research Institute, Toronto, Ontario, Canada; Lunenfeld-Tanenbaum Research Institute, Toronto, Ontario, Canada; Lunenfeld-Tanenbaum Research Institute, Toronto, Ontario, Canada; Lunenfeld-Tanenbaum Research Institute, Toronto, Ontario, Canada; Lunenfeld-Tanenbaum Research Institute, Toronto, Ontario, Canada; Sunnybrook Health Sciences Centre, toronto, Ontario, Canada; Michael Garron Hospital, Toronto, Ontario, Canada; University of Toronto, Toronto, Ontario, Canada; Lunenfeld-Tanenbaum Research Institute, Toronto, Ontario, Canada; Mt. Sinai Hospital, Toronto, Ontario, Canada

## Abstract

**Background:**

The protection afforded by COVID-19 vaccination wanes over time and varies by SARS-CoV-2 strain. Identifying a marker to serve as an immunologic correlate of protection (CoP) is crucial to ensure future vaccine iterations are sufficiently protective and to inform recommendations for the timing of subsequent vaccine doses.

**Figure 1.**
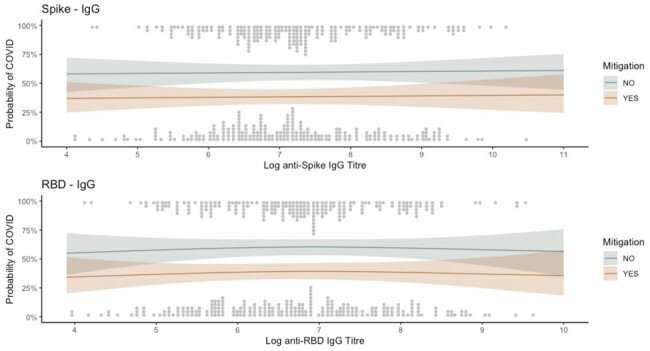
Antibody titres. The probability of COVID-19 by log transformed anti-Spike and anti-RBD IgG titres with raw values represented by dots.

**Methods:**

A prospective cohort of asymptomatic adults exposed to a household member with COVID-19 was created in Toronto, Ontario from October 1, 2021 until February 21, 2023. At enrolment, anti-spike and anti-receptor binding domain (RBD) IgG titres were measured as were pseudoneutralization titres against wild-type, BA.1 and BA.5 strains for those who provided serum. Participants were followed for 28 days with molecular or antigen testing performed if COVID-19 symptoms arose. A convalescent DBS was collected to assess for asymptomatic infection to compare acute and convalescent anti-nucleoprotein IgG titres. The probability of contracting COVID-19 was modeled using a binomial generalized additive model with a logit link and a thin plate spline on log transformed antibody titres adjusted for whether mitigation measures were employed.

**Figure 2.**
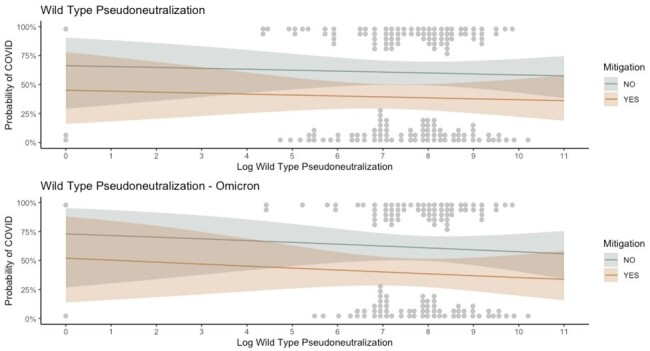
Pseudoneutralization Titres. The probability of COVID-19 by log transformed SARS-CoV-2 wild-type pseudoneutralization antibody titres for the entire cohort and households with a circulating Omicron variant.

**Results:**

In total, 432 individuals from 311 households participated, of which 210 developed COVID-19. The median age was 43 (interquartile range 38-50), 67% (288/432) received 3 vaccine doses, 65% (280/432) were a parent of the index case and 51% (222/432) employed a mitigation measure. There was no difference in the probability of infection by Spike or RBD titres (Figure 1). The difference in probability of COVID for Spike IgG between the 90th (8.83) and 10th percentiles (5.76) is 1.01 (95% CI 0.9 - 1.15, p = 0.84). For RBD IgG the difference between 90th (8.17) and 10th percentiles (5.27) is 1.01 (95% CI 0.89 - 1.14, p = 0.87). Wild-type pseudoneutralization titres also did not confer protection (Figure 2). No differences were identified by circulating household SARS-CoV-2 variant or by pseudoneutralization strain.

**Conclusion:**

Vaccine derived immune markers of asymptomatic household contacts do not correlate with protection against COVID-19. This could be due to the nonexistence of humoral correlates, rapid rise in antibody titres during the pre-symptomatic phase, or changes in behaviour overrides immune protection.

**Disclosures:**

Allison McGeer, MD, AstraZeneca: Honoraria|GSK: Honoraria|Merck: Honoraria|Moderna: Honoraria|Novavax: Honoraria|Pfizer: Grant/Research Support|Pfizer: Honoraria|Roche: Honoraria|Seqirus: Grant/Research Support|Seqirus: Honoraria

